# Effects of dexmedetomidine on A549 non-small cell lung cancer growth in a clinically relevant surgical xenograft model

**DOI:** 10.1038/s41598-023-39704-3

**Published:** 2023-08-01

**Authors:** Ji Hae Jun, Jae-Kwang Shim, Ju Eun Oh, Kwang-Sub Kim, Young-Lan Kwak, Sarah Soh

**Affiliations:** 1 Anesthesia and Pain Research Institute, Yonsei University College of Medicine, Seoul, Republic of Korea; 2 Department of Anesthesiology and Pain Medicine, Yonsei University College of Medicine, 50 Yonsei-ro, Seodaemun-gu, Seoul, 03722 Republic of Korea

**Keywords:** Lung cancer, Cancer microenvironment

## Abstract

The perioperative milieu following curative lung cancer surgery is accompanied by a stress response. Inflammasomes mediate inflammation resulting in the unfavorable immunomodulation of natural killer (NK) cell activity, thus promoting cancer progression. This study aimed to investigate the effects of dexmedetomidine (DEX) on the innate immune system, chronic inflammation, and lung cancer progression in a clinically relevant human-to-mouse xenograft model. The human lung cancer cell line A549-luc was subcutaneously injected into BALB/c nude mice. Saline or dexmedetomidine was administered for 2 weeks via an implanted osmotic minipump. After 4 weeks, the tumor size and weight were measured. NK cell activity, serum interferon-γ, interleukin (IL)-1β and tumor necrosis factor (TNF)-α levels were also measured. IL-10, IL-18, and inflammasome expression levels were assessed in the tumor tissues. DEX caused a decrease in tumor size, tumor weight, and IL-1β and TNF-α levels and an increase in NK cell activity and IFN-γ level. IL-10 and IL-18 expression was significantly decreased in the DEX-treated group. NLRP3, CTP1A, TXNIP, ASC, IL-1β, and caspase-1 protein levels were decreased in the DEX-treated group. In conclusion, the use of DEX for 2 weeks inhibited lung cancer progression by suppressing inflammasome- and IL-1β signaling-induced inflammation and enhancing NK cell activity.

## Introduction

Although surgery is the first treatment option for lung cancer, micrometastases and tumor cell dislodgement during surgical manipulation are the major risks. Even after surgery, tumor cells disseminate into the vascular and lymphatic systems, initiating tumor regrowth and recurrence^1^. The perioperative milieu is inevitably associated with surgery-induced stress responses, such as inflammation, resulting in unfavorable immunomodulation of natural killer (NK)-cell activity and cellular immunity, thus inhibiting the primary defense mechanism against cancer dissemination^2^. Simultaneously, pro-inflammatory signals in malignant tissues intensify to promote tumor growth and prolong pathological conditions^3,4^. Emerging evidence supports the pivotal role of aberrantly activated inflammasomes, including NLRP3 (nucleotide-binding oligomerization domain, leucine-rich repeat and pyrin domain-containing protein 3), in lung cancer pathogenesis^5,6^. In addition to the induction of potent inflammatory cytokines, NLRP3 also suppresses NK cell-associated cancer immunity in various lung metastasis models^7^.

Dexmedetomidine (DEX), a highly selective α_2_-adrenergic agonist, has several characteristics that inhibit the inadvertent formation of a pro-tumoral environment after surgery^8^. DEX promotes anti-inflammatory environment, attenuates stress response, and preserves immune cytotoxicity^9,10^. These effects have been validated in few randomized controlled trials on patients who underwent surgery for breast or colon carcinomas^11,12^. Considering that the post-surgery pro-tumoral environment persists for approximately 2 weeks^4,13,14^, the long-term use of DEX may induce favorable modulations in the inflammatory and immune responses. However, to the best of our knowledge, no research has been conducted in this regard.

In this study, we aimed to investigate the effects of a 2-week DEX regimen on inflammation, immunosuppression, and tumor growth in a clinically relevant xenograft mouse lung cancer model.

## Results

### DEX inhibits the growth of lung cancer xenografts

**Figure 1:**
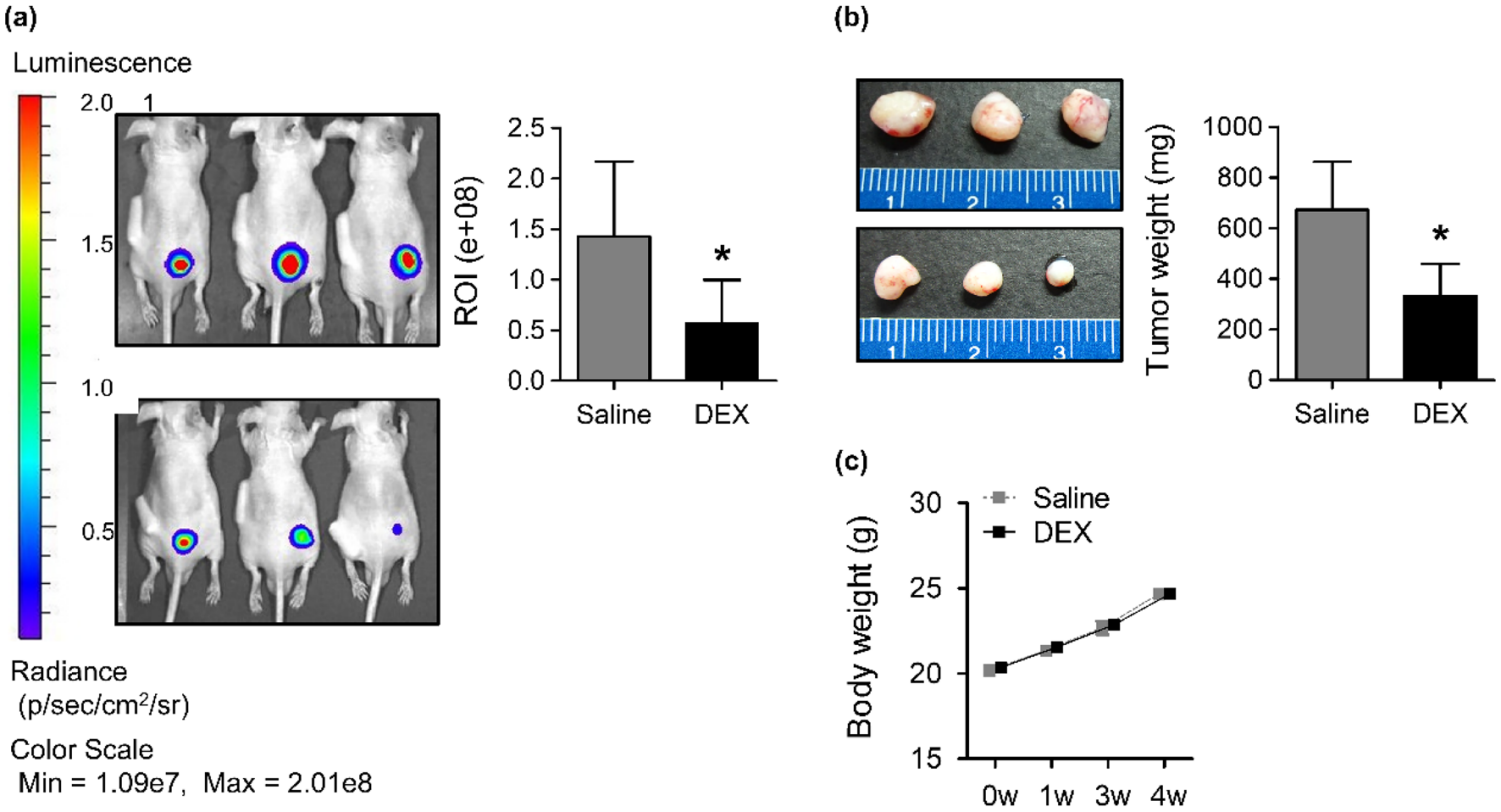
Effect of DEX on tumor growth in nude mice. (**a**) The tumor volumes investigated using the IVIS spectrum and representative images visualized by luciferase expression. (**b**) Representative images and wet weights of the excised tumors. (**c**) Body weight changes. *P < 0.05. *ROI* regions of interest, *DEX* dexmedetomidine, *IVIS* in vivo imaging system.

Subcutaneous injections of A549-luc cells promoted tumor development from as early as 7 days. At the end of 4 weeks, in vivo imaging system (IVIS) revealed significant suppression of tumor volume in DEX-treated nude mice (*P* < 0.05, compared with mice in the control saline-treated group). The intensity of fluorescence from low to high (blue to red) indicated an increasing tumor burden, as calculated by the regions of interest (ROI) (Fig. 1a). The size and wet weights of the excised tumors were lower in the DEX-treated group than in the saline-treated group (Fig. 1b). The body weights of the saline- and DEX-treated groups were similar throughout the study period (Fig. 1c).

### DEX increased NK cell activity

**Figure 2:**
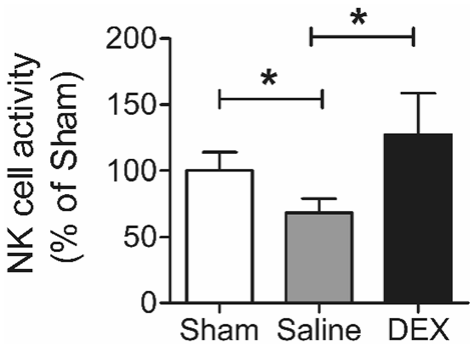
NK cell activity detected using the NK Vue kit assay. *P < 0.05. *DEX* dexmedetomidine, *NK cell* natural killer cell.

NK cell activity was 30% lower in the saline-treated group (68 ± 11%) than in the normal-sham group (100 ± 14%). In contrast, NK cell activity was significantly higher in the DEX-treated group than in the saline-treated group (68 ± 11% vs. 127 ± 31%, *P* < 0.05) (Fig. 2).

### DEX stimulated IFN-γ expression and inhibited IL-1β and TNFα expression

**Figure 3:**
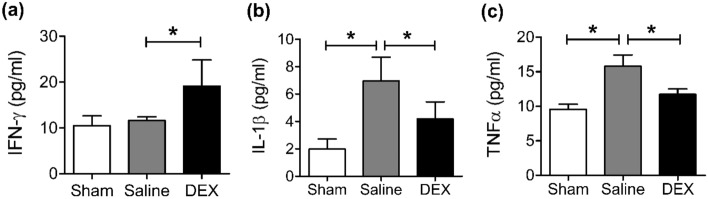
Serum cytokine concentrations of (**a**) IFN-γ, (**b**) IL-1β, and (**c**) TNFα detected using ELISA. *P < 0.05. *DEX* dexmedetomidine, *IFN-γ* interferon-γ, *IL-1β* interleukin-1β, *TNF-α* tumor necrosis factor-α, *ELISA* enzyme-linked immunosorbent assay.

To determine the effect of DEX on inflammatory cytokines in A549-luc xenograft models, serum interferon (IFN)-γ, interleukin (IL)-1β, and tumor necrosis factor (TNF)α levels were analyzed using enzyme-linked immunosorbent assay (ELISA). DEX treatment increased the expression of IFN-γ (11.6 ± 0.8 vs. 19.1 ± 5.7, *P* < 0.05) and decreased that of IL-1β (7.0 ± 1.7 vs. 4.2 ± 1.3, *P* < 0.05) and TNFα (15.8 ± 1.6 vs. 11.7 ± 0.8, *P* < 0.05) compared with saline treatment (Fig. 3).

### DEX suppressed IL-10 and IL-18 mRNA expression

**Figure 4:**
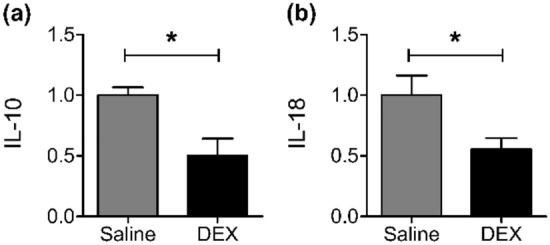
mRNA expression of (**a**) IL-10 and (**b**) IL-18 in A549 tumor tissues by real-time quantitative polymerase chain reaction. *P < 0.05. *DEX* dexmedetomidine, *IL* interleukin.

The mRNA expression of IL-10 and IL-18 in tumor tissues was assessed by *quantitative polymerase chain reaction (*qPCR). Compared with saline treatment, DEX treatment caused a significant reduction in the mRNA levels of IL-10 (1.0 ± 0.06 vs. 0.5 ± 0.14, *P* < 0.05) and IL-18 (1.0 ± 0.16 vs. 0.55 ± 0.09, *P* < 0.05; Fig. 4).

### DEX decreased inflammasome protein levels

**Figure 5:**
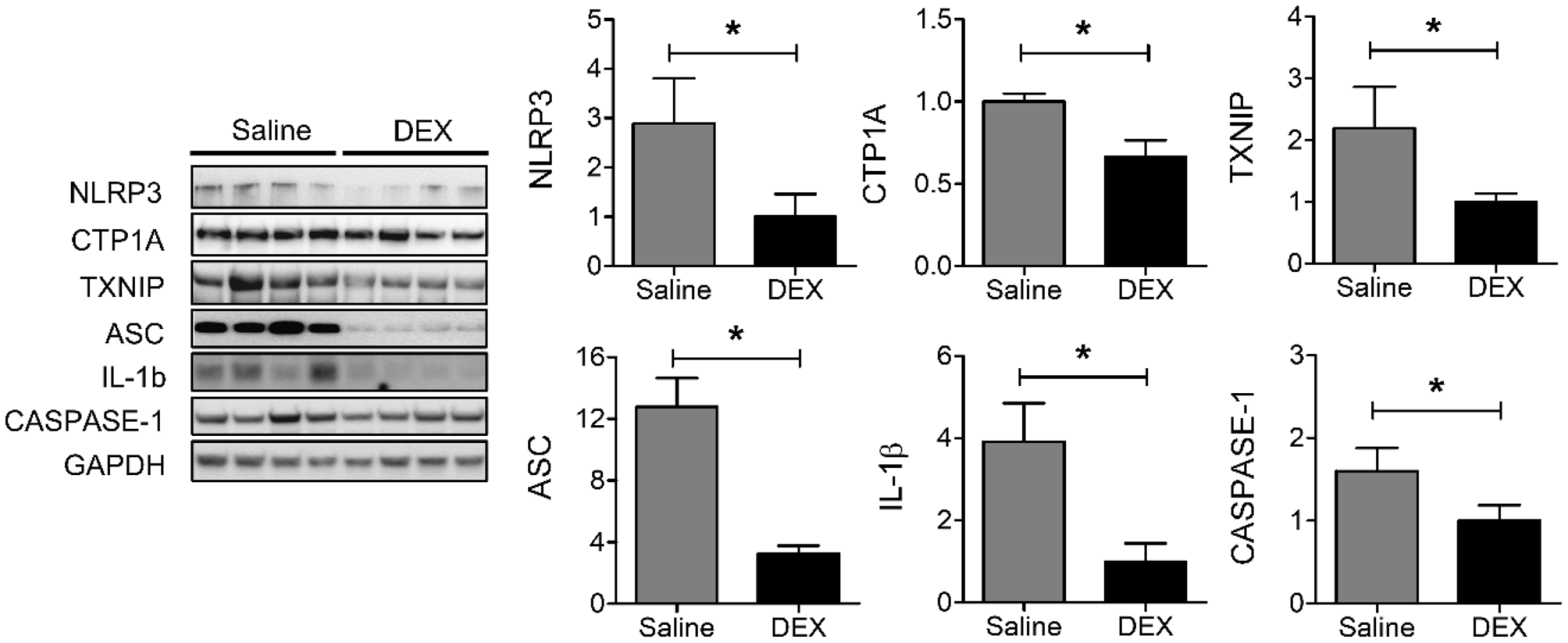
The protein expression levels of NLRP3, CTP1A, TXNIP, ASC, IL-1β, and caspase-1 in A549-tumor tissues. Each western blot is the result of a different gel and has been grouped. The uncropped blots are presented in Supplementary Fig. S1 and some membranes were cut prior to hybridization with antibodies. *P < 0.05. *DEX* dexmedetomidine, *NLRP3* nucleotide-binding oligomerization domain (NOD)-like receptor with a pyrin domain 3, *TXNIP* thioredoxin-interacting protein, *ASC* apoptosis-associated speck-like protein, interferon-γ, *IL-1β* interleukin-1β.

DEX treatment caused a significant reduction in the protein levels of NLRP3 (2.9 ± 0.9 vs. 1.0 ± 0.5, *P* < 0.05), CTP1A (1.0 ± 0.05 vs. 0.7 ± 0.1, *P* < 0.05), thioredoxin-interacting protein (TXNIP) (2.2 ± 0.7 vs. 1.0 ± 0.1, *P* < 0.05), apoptosis-associated speck-like protein containing a caspase recruitment domain (ASC) (12.8 ± 1.9 vs. 3.3 ± 0.5, *P* < 0.05), IL-1β (3.9 ± 0.9 vs. 1.0 ± 0.4, P < 0.05), and caspase-1 (1.6 ± 0.3 vs. 1.0 ± 0.2, *P* < 0.05) compared with saline treatment (Fig. 5).

## Discussion

In a clinically relevant human-to-mouse lung cancer xenograft model, we observed that DEX administration for 2 weeks after surgical stimulation inhibited tumor growth. By promoting NK cell activity and IFN-γ expression, DEX treatment resulted in the favorable modulation of the hostile postsurgical milieu that facilitates tumor growth. DEX treatment also decreased the levels of pro-inflammatory cytokines, including IL-1β, TNFα, IL-10, and IL-18, which are major tumor growth promoters. In the tumor tissue, DEX treatment significantly reduced the expression of CTP1A and TXNIP, which are involved in reactive oxygen species (ROS)-related regulatory mechanisms associated with NLRP3 inflammasome activation. Consequently, the expression of NLRP3, ASC, and caspase-1, which comprise the NLRP3-inflammasome complex known to suppress NK cell activity, was significantly reduced. Further, a significant decrease was noted in IL-1β levels in the tumor tissue.

In the lung cancer xenograft model, tumor volume after 4 weeks was significantly lower in the DEX-treated group than in the saline-treated group. Compared with the normal-sham group, NK cell activity decreased and IFN-γ levels were unchanged in the saline-treated group. Compared with the saline group, significant increases in NK cell activity and serum IFN-γ levels were observed in the DEX-treated group. These results are consistent with those of previous studies, which demonstrated that both NK cell cytotoxicity and IFN-γ secretion were consistently and profoundly suppressed during the perioperative period^15,16^ and in patients with lung cancer^17^. NK cells are crucial regulators of tumor cell survival^18^. However, tumor cells circumvent NK cell-mediated surveillance by cultivating an immunosuppressive microenvironment, leading to dysfunction of NK cells^19^. It is also known that NK cells are unable to prevent tumor promotion or progression due to attenuated cytotoxicity in the lung cancer microenvironment^20^. Similarly, the present study noted a decline in NK cell activity in the control group, which received cancer cell injections, in comparison to the sham group. Furthermore, in the DEX group, NK cell activity significantly increased compared to the control group. NK cells contribute to antigen-specific adaptive immunity by producing IFN-γ, which promotes Th1 response^21^. IFN-γ signaling in tumor cells enhances tumor immunogenicity, promotes infiltration of mononuclear cells into tumor tissue, and inhibits tumor angiogenesis^22^. Therefore, it seems cogent to assume that increased NK cell activity and serum IFN-γ levels due to administration of DEX inhibited tumor growth.

Serum concentrations of the pro-inflammatory cytokines TNFα and IL-1β were greater in the saline-treated group than in the normal-sham group and were attenuated by DEX treatment. Under normal circumstances, the acute inflammatory response elicited by surgical stimulation is self-limiting^23^. However, intensified pro-inflammatory signals are sustained in malignant tissues, to support tumor proliferation^3,24^. TNF-α is a potent immunostimulatory cytokine and contributes to maintenance of a pro-inflammatory environment^25^. TNF-α plays an important role in tumor proliferation and metastasis^26^; IL-1β is known to play a critical role in tumor progression^27^. Abnormally high concentrations of TNFα in tumors have been confirmed in several studies, suggesting the involvement of this cytokine in preneoplastic lesions and malignant diseases^28^. IL-1 has been shown to promote tumor growth and metastasis in transplanted mouse and human tumors, and IL-1β has been shown to be important drivers of carcinogenesis and metastasis^29^. Increased serum concentrations for both these cytokines have been described in lung cancer^27,30^. Consistent with these findings, our study also observed a significant elevation in serum levels of both cytokines in the control group, in comparison to the sham group. Therefore, our findings suggest that DEX inhibited lung cancer growth by modulating the levels of these inflammatory mediators.

The cytokine balance of Th1/Th2 is important in anti-tumor immunity, with Th2-predominant ratios being associated with lung cancer^31^. We observed that IL-10 and IL-18 expression in tumor tissue was significantly decreased in the DEX-treated group compared with the saline-treated group. Typically, IL-10 is classified as a Th2 cytokine, and IL-18 stimulates Th2 immune responses^32^. In addition, the crucial roles of IL-18 in upregulating tumorigenic, angiogenic, and prometastatic mechanisms in cancer cells have been reported^33^. Thus, the decreased expression of these Th2 cytokines due to DEX administration may have contributed to the inhibition of cancer growth.

NLRP3, a cytoplasmic multiprotein complex, upregulates the maturation and release of inflammatory cytokines, such as IL-1β and IL-18^34^, and suppresses the activation of NK cells that secrete IFN-γ^7^. Although the NLRP3 inflammasome appears to have both pro- and anti-tumorigenic effects in a context-dependent and tissue-specific manner, several experimental studies have reported that it enhances lung cancer progression^5,35^. Among other mechanisms, excessive production of ROS by inflammatory cells in the tumor microenvironment is important for activating the NLRP3 inflammasome^36^. ROS can cause oxidative damage to mitochondria^37^, in turn activating the inflammasome. In this study, DEX decreased the expression of NLRP3, ASC, and caspase-1, constituting the inflammasome complex, as well as of CTP1A and TXNIP, which are related to inflammasome activation^38,39^. These results could be associated with the decrease in IL-1β expression in tumor tissue and increase in NK cell activity by DEX.

Some researchers have noted that DEX could promote tumor growth via activation of α2‑adrenoreceptors (expressed on cancer cells or host stromal cells) or imidazoline receptor, which increases the formation of hypoxia-inducible factor and vascular endothelial growth factor involved in the proliferation and growth of non-small cell lung cancer cells^40–42^. Whether DEX definitively enhances or inhibits tumor growth remains to be elucidated; differences in the experimental design, cancer histologic cell type, DEX administration schedule, and DEX dosage could affect the outcome. The duration of DEX infusion is likely to be responsible for the adverse outcomes, as it was relatively short in studies showing cancer-growth-promoting effects^41,42^. In addition, the deleterious effects of DEX on tumor progression seemed to be dose-dependent^42^. In a previous study of DEX favoring tumor growth^42^, rats received a dosage of 5 µg/kg/hour (120 µg/kg/day), which did not result in a 50% reflex loss in any of the whisker, righting, or startle reflexes. In that study, however, rats showed behavioral signs such as reduced movement and delayed responses compared to naïve peers. This dosage of 120 µg/kg/day appears to fall within the range of plasma concentrations clinically used for hypnotic effects. Conversely, in the current study, a subhypnotic dose (20 μg/kg/day) was administered over a longer duration, which appears to hold potential for clinical application.

This study has some limitations associated with our study design. First, the generalizability of the findings to human lung cancer progression is uncertain, as is the case for all tumor models used in translational studies. Second, concomitant in vitro studies may have provided more insight into the exact causality of the observed results. However, in vitro studies were precluded as the influence of DEX requires intact innervation of the autonomic nervous system, necessitating in vivo models.

In conclusion, our study provides empirical evidence for the beneficial influence of the long-term use of DEX in modulating the tumor microenvironment in a clinically relevant human-to-mouse xenograft model of lung cancer. Acute and chronic inflammatory responses and immunosuppression induced by the tumor itself or noxious stimulation were attenuated with the long-term use of DEX, thereby inhibiting tumor growth. Our study emphasizes the importance of major inflammatory mediators in lung cancer growth and identified the activation of the NLRP3 inflammasome as an important mechanism and potential therapeutic target for cancer treatment. To validate the effects of DEX on inflammation and immunosuppression, future studies should assess the effects of different treatment doses and durations in various cancer types.

## Methods

### Animal model and ethical approval

All experimental procedures in this study were approved by the Institutional Animal Care and Use Committee of Yonsei University Health System (IACUC 2018-0149) and conducted in accordance with all guidelines and regulations. And this study is reported in accordance with ARRIVE guidelines (https://arriveguidelines.org). Six-week-old male BALB/c nude mice (n = 31) weighing 17–18 g were housed and tested in accordance with the Guide for the Care and Use of Laboratory Animals (US National Institutes of Health). No procedural mortalities were recorded.

### Cell culture and lentiviral particle transduction

Human non-small cell lung cancer cell line A549 was purchased from the American Type Culture Collection (Manassas, VA, USA). A549 cells were maintained in Roswell Park Memorial Institute (RPMI) supplemented with 10% heat-inactivated fetal bovine serum (FBS), 100 U/mL penicillin, and 100 µg/mL streptomycin. To induce A549-luc cells, A549 cells (5 × 10^4^) were cultured in a 24-well plate before viral infection and incubated overnight with a complete optimal medium (0.5 mL) containing serum and antibiotics. One day after, the medium was removed and replaced by 0.5 mL of complete medium with 4 µg/mL polybrene. A day later, cells were infected by adding 50 µL of Redifect lentiviral particles (20 µL PerkinElmer, Waltham, MA, USA) to the culture. Stable clones of A549-luc cells were selected using 5 µg/mL puromycin dihydrochloride. Transduction efficiency was determined using an optical IVIS spectrum (PerkinElmer).

### Xenograft models and study groups

The mice were randomly assigned to three groups: (i) normal-sham group (n = 7), (ii) A549-luc-saline (n = 12), and (iii) A549-luc-DEX (n = 12). Tumor xenograft models were established by subcutaneous injection of A549-luc cells (5 × 10^6^ in 0.1 mL PBS) into the right flank of BALB/c nude mice. To evaluate the therapeutic response of the metastatic lung tumor xenografts to DEX, 24 mice who received subcutaneous implantation of A549-luc cells were randomly assigned to either saline or DEX (20 μg/kg/day) treatment using a micro-osmotic pump system (model 1002, ALZET, Cupertino, CA, USA). The pump flow rate was set to 0.25 μL/h, and the treatments were delivered continuously for 14 days. Pumps were surgically implanted subcutaneously into the left flank of BALB/c nude mice after isoflurane anesthesia simultaneously with the tumor cell implantation. The normal-sham group underwent only a skin incision. Four weeks after cell implantation, imaging was performed using the IVIS, nude mice were euthanized, and tumors were excised and weighed.

### In vivo bioluminescence imaging

Images were recorded using the IVIS spectrum and analyzed using Living Image 4.5.5 software (PerkinElmer). D-luciferin (potassium salt, PerkinElmer) was injected intraperitoneally at 150 mg/kg before bioluminescence imaging to generate bioluminescence signals. Animals were anesthetized with isoflurane and placed in the imaging chamber. All fluorescence images were acquired with a 7-m exposure. For quantitative comparison, ROIs were drawn over the tumor, and the results are expressed as the mean ± standard deviation (n = 7).

### Natural killer cell activity and ELISA

Serum was collected from the A549-luc xenograft mouse model at the time of euthanasia. The cytotoxic activity of NK cells was determined using an NK Vue Gold kit (ATGen, Seongnam, Korea), according to the manufacturer’s instructions. Serum levels of IFN-γ, IL-1β, and TNFα were analyzed using ELISA commercial kits (R&D Systems, Minneapolis, MN, USA) according to the manufacturer’s instructions.

### Real-time quantitative polymerase chain reaction (qPCR)

IL-10 and IL-18 mRNA expression levels were assessed by qPCR using a SensiFAST SYBR Hi-ROX kit (Bioline USA, Taunton, MA, USA) and AB7500 Fast qPCR System (Applied Biosystems, Foster City, CA, USA). Seven tumor tissues per group were analyzed in quadruplicate. Target genes were normalized to the reference housekeeping gene GAPDH. Fold differences were calculated for each group using the normalized CT values for the normal-sham group. The primers used are listed in Supplementary Table S1.

### Western blot analysis

The levels of inflammasome protein expression were assessed using western blot analysis. Five tumor tissues per group were analyzed in triplicate. Protein concentrations were measured using the Bradford assay (Thermo Fisher Scientific, Carlsbad, CA, USA), and equal amounts of protein from each sample were subjected to western blot analysis with anti-TXNIP (Santa Cruz Biotechnology, Dallas, TX, USA), anti-caspase-1, GAPDH (Cell Signaling Technology, Beverly, MA, USA), anti-NLRP3, anti-CTP1A, anti-ASC, and anti-IL-1β (Abcam, Cambridge, UK). Target proteins were normalized to the reference housekeeping gene GAPDH.

### Statistical analysis

All results are expressed as mean ± standard deviation. Statistical analyses were performed using Student’s *t*-test, one-way analysis of variance (ANOVA) followed by Tukey’s multiple comparison test, or repeated-measures ANOVA. Statistical significance was set at *P* < 0.05.

## Supplementary Information

Supplementary Information.

## Data Availability

The datasets used or analyzed during the current study are available from the corresponding author upon reasonable request.
