# Supplementary material for: Effects of dexmedetomidine on A549 non-small cell lung cancer growth in a clinically relevant surgical xenograft model
Source: Sci Rep. 2023 Aug 1;13:12471. doi: 10.1038/s41598-023-39704-3 (PMC10393998; doi:10.1038/s41598-023-39704-3)
Supplement: Supplementary file 1 — Supplementary Information. [file 41598_2023_39704_MOESM1_ESM.pdf]

Supplementary information

Article title: Effects of dexmedetomidine on human non-small cell lung cancer growth in a clinically relevant surgical xenograft model

Journal name: Scientific Reports

Author names: Ji Hae Jun, Jae-Kwang Shim, Ju Eun Oh, Kwang-Sub Kim, Young-Lan Kwak, and Sarah Soh

Correspondence: Sarah Soh, MD, Ph.D.

E-mail: yeonchoo@yuhs.ac

Department of Anaesthesiology and Pain Medicine, Yonsei University College of Medicine, 50 Yonsei-ro, Seodaemun-gu, Seoul 03722, Republic of Korea

Telephone: 82-2-2228-8500

Fax: 82-2-364-2951

ORCID ID: 0000-0001-5022-4617

SUPPLEMENTARY TABLES

Supplementary Table S1 2

Supplementary Figure S1 3

Supplementary Table S1. The primers for quantitative polymerase chain reaction (qPCR) assay

Gene	Primer names	Primer sequence (5′ to 3′)
IL-10	Forward	GGTGAGAAGCTGAAGACCCT
IL-10	Reverse	ACACCTTGGTCTTGGAGCTT
IL-18	Forward	GGACACTTTCTTGCTTGCCA
IL-18	Reverse	ACCCTCCCCACCTAACTTTG
GAPDH	Forward	CTGGAGAAACCTGCCAAGTA
GAPDH	Reverse	AGACAACCTGGTCCTCAGTG

Supplementary Figure S1. Original images of Western blot (Fig 5)

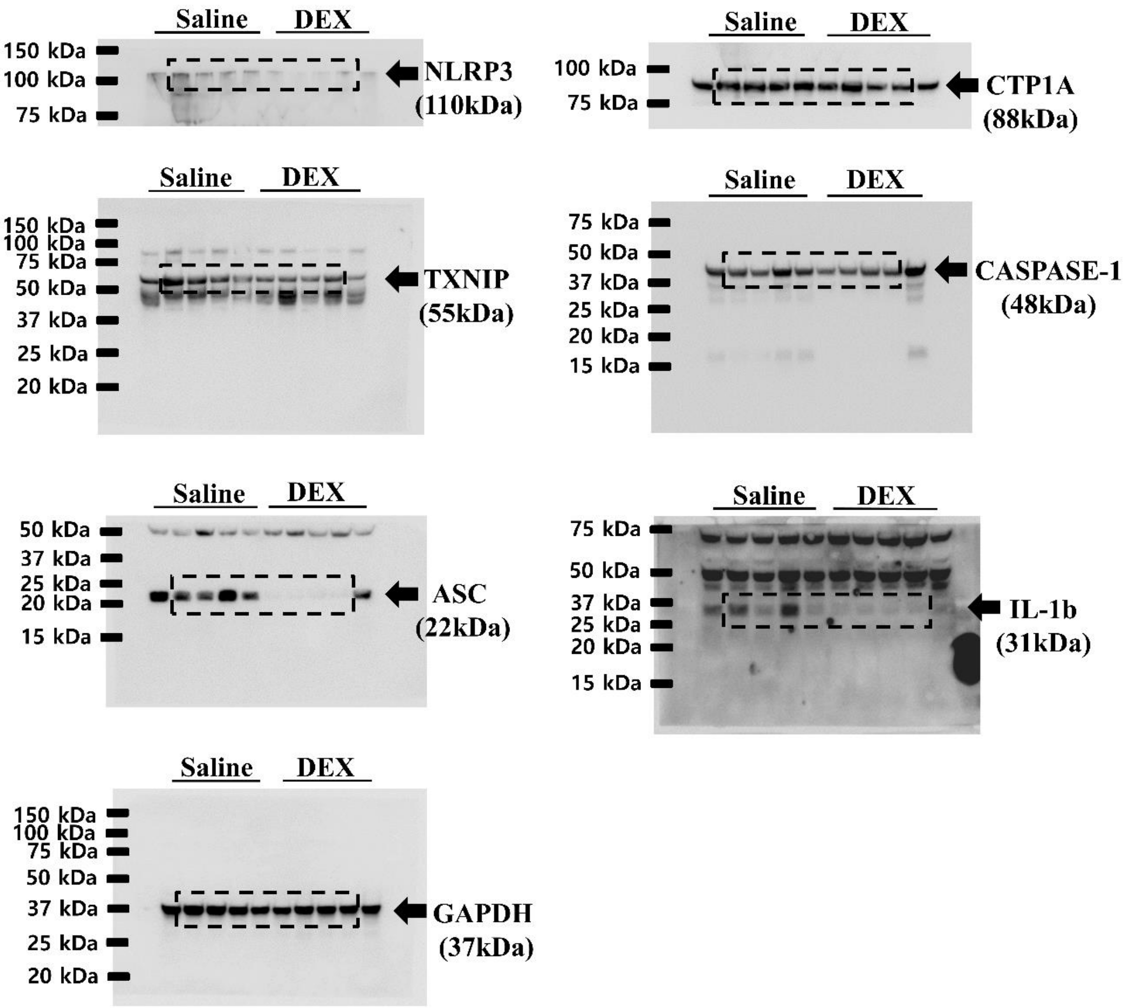

Lanes 1 to 5 correspond to samples from the A549-luc-saline group, and lanes 6 to 10 correspond to samples from the A549-luc-DEX group. Some membranes were cut prior to hybridization with antibodies. Lanes 2 to 9 (the dotted squares) are shown in the manuscript for publication.
